# Prevalence and Determinants of Nomophobia Among Healthcare Workers in Nepal: A Cross-Sectional Study

**DOI:** 10.7759/cureus.96570

**Published:** 2025-11-11

**Authors:** Prakash Banjade, Asmita Itani, Munish Sharma, Reena Shah, Jasmit Shah, Aakansha Singh, Rahul Kashyap, Salim Surani

**Affiliations:** 1 Pediatrics, Brookdale University Hospital Medical Center, Brooklyn, USA; 2 General Medicine, Tribhuvan University Institute of Medicine, Kathmandu, NPL; 3 Pulmonary and Critical Care, Baylor Scott & White Medical Center - Temple, Temple, USA; 4 Medicine, Aga Khan University, Nairobi, KEN; 5 General Medicine, Global Remote Research Scholars Program, Saint Paul, USA; 6 Medicine, Drexel University College of Medicine, Philadelphia, USA; 7 Global Clinical Scholars Research Training, Harvard Medical School, Boston, USA; 8 Research, Global Remote Research Scholars Program, Saint Paul, USA; 9 Critical Care Medicine, Mayo Clinic, Rochester, USA; 10 Medicine, Valley Coastal Bend Veterans Affairs, Harlingen, USA; 11 Medicine, University of Houston, Houston, USA; 12 Anesthesiology, Mayo Clinic, Rochester, USA

**Keywords:** cross-sectional study, healthcare workers, mental health, nomophobia, smartphone

## Abstract

Introduction: Nomophobia, the fear of being without a mobile phone, has become a significant concern in the current digital age. Healthcare professionals rely heavily on mobile devices for communication and information access. This cross-sectional study aimed to assess the prevalence and determinants of nomophobia among healthcare workers in Nepal.

Methods: A modified Nomophobia Questionnaire (NMP-Q) was used to determine nomophobia scores. Demographic characteristics such as age, gender, race, type of smartphone, hours spent on a smartphone, and type of medical specialty were considered to evaluate nomophobia in diverse groups of healthcare workers. Summary statistics were presented as frequencies and percentages, and all group comparisons were done using Fisher's exact test. All analyses were conducted using SPSS version 20 (IBM Corp., Armonk, NY).

Results: A total of 519 responses were collected. Gender and age were significantly associated with nomophobia levels. For nomophobia level 1, females exhibited 72.4% (n = 63) severe and 54.7% (n = 196) moderate levels, compared to 27.6% (n = 24) and 45.3% (n = 162) for males (p = 0.002). For nomophobia level 2, 71.4% (n = 60) of females had severe levels versus 28.6% (n = 24) of males (p = 0.009). A total of 55.2% (n = 48) of severe nomophobia level 1 cases were aged 18-25 years, and 37.9% (n = 33) were aged 26-35 years (p = 0.008). Similarly, for nomophobia level 2, 47.6% (n = 40) were aged 18-25 years and 45.2% (n = 38) were aged 26-35 years (p = 0.022).

Conclusions: The study revealed a considerable prevalence of nomophobia among healthcare workers in Nepal, with a significant impact on specific demographic groups, particularly females and individuals aged 18-25 years.

## Introduction

With the widespread availability of the internet via smartphones, digital access has become an integral part of healthcare professionals' work [1]. The increasing reliance on mobile devices has given rise to "nomophobia." Nomophobia is a fear or anxiety related to being without a mobile phone or being unable to use it for any reason [2]. It is a psychological condition in which one fears being disconnected from mobile phone connectivity. The term is derived from a combination of "no-mobile-phone phobia" and is classified in the Diagnostic and Statistical Manual of Mental Disorders, Fourth Edition (DSM-IV) criteria and categorized as "phobia for particular or specific things" [2]. People with nomophobia may experience physical symptoms of anxiety when they are separated from their phones or unable to use them. The condition can interfere with daily life and may require treatment. Despite the possible consequences, studies on nomophobia among healthcare providers are limited. This study, conducted in Nepal, was part of a Global Providers' Phone-lessness Phobia (Global 3-P) study. This study explored the prevalence and degree of this specific type of phobia among healthcare practitioners.

## Materials and methods

Study design

As part of the Global 3-P study [3], we conducted a cross-sectional survey among healthcare professionals in Nepal from April 1 to July 1, 2023. Our team distributed a self-reported modified Nomophobia Questionnaire (NMP-Q) to the participants via a web-based survey that included a series of questions for data collection purposes. The study complied with the Strengthening the Reporting of Observational Studies in Epidemiology (STROBE) guidelines [4].

Sample size

This study is part of the Global 3-P project, which calculated a minimum required sample size of 9,379 participants to achieve adequate statistical power (95% confidence interval, 1% margin of error). The Nepalese subset consisted of 519 healthcare students and professionals who completed the survey. As the Nepal data formed part of the larger global dataset, a separate sample size calculation was not conducted.

Data collection

A modified version of the NMP-Q was used to assess nomophobia scores [5]. The final modified questionnaire comprises 33 questions, categorized into sections encompassing 25 questions and eight demographic details to explore the phenomenon of nomophobia. The six subsections consisted of being unable to access information, losing connections, being unable to communicate, giving up convenience, daily habits, and the type of smartphone used, as well as the hours spent on the smartphone for both personal and work-related activities. Level I nomophobia is assessed by 14 refined questions (categories A-D), which were produced by condensing and refining the original 20-item NMP-Q [5]. Level II nomophobia is assessed by 22 questions, consisting of the 14 refined items from level I plus eight additional items (category E). Based on the nomophobia score, each level can be categorized as mild, moderate, or severe.

Survey validation

The modified survey underwent a rigorous two-phase validation process. Seven researchers conducted the internal validation, which included reviewing the instrument for clarity and coherence. External validation was performed by eight psychiatrists, whose feedback led to refinements in sections E and F and the division of the “Total Hours Spent on a Smartphone” question into separate items for personal and work-related use. To enhance readability, emotionally descriptive terms (e.g., “UNCOMFORTABLE,” “ANNOYED,” “WORRIED,” “ANXIOUS”) were capitalized for emphasis. Both content and construct validity were confirmed. Content validity ensured the questions adequately represented the domain of nomophobia, with expert input from psychiatry, technology, and healthcare professionals. Construct validity confirmed internal consistency among items reflecting psychological dependence on smartphones.

Statistical analysis

All statistical analyses were conducted using SPSS version 20 statistical software (IBM Corp., Armonk, NY). Summary statistics were presented as frequencies and percentages. All group comparisons were evaluated using Fisher's exact test. Our hypothesis considered gender (male, female, or other) and the number of hours spent on smartphones for personal/work activities as primary predictors. Statistical significance was defined by a two-tailed p < 0.05.

Ethical consideration

Participation in the study was voluntary, and individuals who chose to participate provided informed consent by completing the survey. No personal or institutional identifiers were collected or disclosed during the study. The Mayo Clinic Institutional Review Board granted an exemption from the review of the study proposal.

## Results

A total of 519 responses were collected from 1 April 2023 to 1 July 2023. Among them, 238 (45.90%) were aged 18-25 years, and 209 (40.30%) were 26-35 years. More than half (293, 56.50%) of the respondents were females, and 497 (95.8%) participants identified as South Asians. Smartphone preferences indicated a higher proportion of Android users, with 300 (57.80%) participants using Android phones compared to 210 (40.50%) Apple iOS users. Table 1 summarizes the overall demographic characteristics of the participants. Most participants spent ≤ three hours on smartphones, with 336 (64.70%) used for personal activities and 426 (82.10%) used for work-related activities. This suggests that smartphone usage for work-related activities was higher than for personal ones. Regarding years of experience in the healthcare field, 175 (33.70%) had less than one year of experience, followed by 122 (23.50%) with two to five years of experience.

**Table 1:** Demographic characteristics of the participants.

Demographic characteristics		Participants	
Age	18-25	238	45.90%
	26-35	209	40.30%
	36-45	55	10.60%
	≥46	17	3.30%
Gender	Male	225	43.40%
	Female	293	56.50%
	Other/prefer not to answer	1	0.20%
Race	South Asian	497	95.80%
	Others	22	4.20%
Type of smartphone used	Android	300	57.80%
	Apple iOS	210	40.50%
	Others	9	1.70%
Hours spent on a smartphone			
Personal activities	≤3 hours	336	64.70%
	3.1 - 5 hours	132	25.40%
	5.1 - 7 hours	36	6.90%
	>7 hours	15	2.90%
Work-related activities	≤3 hours	426	82.10%
	3.1 - 5 hours	57	11%
	5.1 - 7 hours	26	5%
	>7 hours	10	1.90%
Years of experience in the healthcare field	<1	175	33.70%
	1-2	36	6.90%
	2-5	122	23.50%
	6-10	101	19.50%
	11-20	70	13.50%
	≥21	15	2.90%
Role at your institution	Administration	14	2.70%
	Advanced registered nurse practitioner (ARNP)	8	1.50%
	Attending physician	58	11.20%
	Auxiliary/support staff	4	0.80%
	Dentist/dental surgeon	52	10%
	Emergency medical transport	3	0.60%
	Fellow in training	4	0.80%
	Medical student	167	32.20%
	Other	108	20.80%
	Pharmacist (PharmD)	5	1%
	Physical therapist	5	1%
	Physician assistant (PA)	5	1%
	Registered nurse (RN)	45	8.70%
	Researcher	11	2.10%
	Resident in training/junior resident	30	5.80%
Specialties - Broad	Administration	12	3.40%
	Anesthesia/pulmonology/critical care	2	0.60%
	Cardiology	4	1.10%
	Clinical research	2	0.60%
	Community medicine/public health/epidemiology	3	0.90%
	Dental	69	19.60%
	Dermatology	6	1.70%
	Emergency medicine	2	0.60%
	Internal medicine and sub-specialties	21	6%
	Laboratory medicine/pathology	24	6.80%
	Nephrology	2	0.60%
	Neurology	1	0.30%
	Obstetrics and gynecology	5	1.40%
	Occupational therapy/physical therapy	6	1.70%
	Ophthalmology/optometry	6	1.70%
	Pediatrics	12	3.40%
	Pharmacology	9	2.60%
	Pre-clinical	6	1.70%
	Psychiatry/psychology	5	1.40%
	Radiology	4	1.10%
	Surgery	12	3.40%
	Other	3	0.90%
	Unknown	136	38.60%

Section A: Not being able to access information

A total of 307 (59.20%) participants strongly agreed or agreed with feeling uncomfortable without constant access to information using their smartphones. Similarly, 296 (57%) strongly agreed or agreed they would find it annoying if they could not look up information on their smartphones when desired. Meanwhile, 249 (48%) of participants strongly disagreed or disagreed with the prospect of being unable to receive news and updates via their smartphones and becoming nervous. A total of 171 (32.90%) strongly agreed or agreed with the feeling of awkwardness when they could not check notifications on their smartphones.

Section B: Losing connections

Only 174 (33.50%) strongly agreed or agreed that running out of battery in their smartphones would scare them, and 108 (20.80%) strongly agreed or agreed to getting panicked if they ran out of credit or monthly data. The dependence on data signals and Wi-Fi connectivity was evident, with 253 (48.70%) participants strongly agreeing or agreeing to constantly check for signals/Wi-Fi networks when unable to connect. The fear of being stranded without smartphone usage was strongly agreed or agreed by 122 (23.50%) respondents. Additionally, 177 (22.50%) of participants strongly agreed or agreed that feeling disconnected from one's online identity without a smartphone induced nervousness.

Section C: Not being able to communicate

A total of 284 (54.70%) participants strongly agreed or agreed that they would feel anxious at the thought of being unable to communicate with family and friends without their smartphones instantly. The concern extended to the worry that family and friends could not reach them, which was strongly agreed or agreed by 394 (75.90%) respondents. Furthermore, 254 (48.90%) participants strongly agreed or agreed that being unable to receive text messages and calls generated nervousness.

Section D: Giving up convenience

Only 128 (24.70%) respondents strongly agreed or agreed that they would feel a sense of weirdness or uncertainty, not knowing what to do if they did not have their smartphones. Additionally, 252 (48.70%) strongly agreed or agreed, wanting to check their phones when they did not have their smartphones.

Section E: Daily habits

More than half (319, 61.50%) strongly agreed or agreed that they prefer to use their smartphones until they fall asleep at night. Additionally, only 61 (11.80%) of the respondents strongly agreed or agreed to wake up at midnight to check notifications. The morning routine revealed a dependence, as 310 (59.70%) strongly agreed or agreed to check their smartphones immediately upon waking up. Smartphone use extended to various activities, with 125 (24.10 %) strongly agreeing or agreeing to use their devices while eating meals, 296 (57%) strongly agreeing or agreeing to spend their breaks on smartphones rather than engaging in physical activities, and 310 (59.70%) strongly agreeing or agreeing to utilize smartphones in the restroom. Furthermore, entertainment choices showcased a preference for smartphone-based relaxation, as 256 (49.30%) strongly agreed or agreed to watch movies or series on their devices rather than exploring hobbies. The final aspect explored the impact of notifications, revealing that 185 (35.60%) of participants strongly agreed or agreed being easily distracted and facing difficulty resisting calls, texts, and emails. Table 2 summarizes the overall characteristics of the five sections listed in the methods section.

**Table 2:** Overall summary of the five sections, consisting of not being able to access information, losing connections, not being able to communicate, giving up convenience, and daily habits.

Section A: Not being able to access information	Strongly disagree/disagree, N (%)	Neutral, N (%)	Strongly agree/agree, N (%)
I would feel UNCOMFORTABLE without constant access to information through my smartphone	93 (17.90%)	119 (22.90%)	307 (59.20%)
I would be ANNOYED if I could not look information up on my smartphone when I wanted to do so	95 (18.30%)	128 (24.70%)	296 (57%)
Being unable to get the news (e.g., happenings, weather, emails, messages, etc.) on my smartphone would make me NERVOUS	249 (48%)	116 (22.40%)	154 (29.70%)
If I did not have my smartphone with me, I would feel AWKWARD because I could not check my notifications for updates from my connections and online networks	155 (29.90%)	193 (37.20%)	171 (32.90%)
Section B: Losing connections			
Running out of battery in my smartphone would SCARE me	164 (31.60%)	181 (34.90%)	174 (33.50%)
If I were to run out of credits or hit my monthly data limit, I would PANIC	264 (50.90%)	147 (28.30%)	108 (20.80%)
If I did not have a data signal or could not connect to Wi-Fi, then I would constantly CHECK to see if I had a signal or could find a Wi-Fi network	104 (20%)	162 (31.20%)	253 (48.70%)
If I could not use my smartphone, I would be AFRAID of getting stranded somewhere	229 (44.10%)	168 (32.40%)	122 (23.50%)
If I did not have my smartphone with me, I would be NERVOUS because I would be disconnected from my online identity	278 (53.60%)	124 (23.90%)	117 (22.50%)
Section C: Not being able to communicate			
If I did not have my smartphone with me-			
I would feel ANXIOUS because I could not instantly communicate with my family and/or friends	101 (19.50%)	134 (25.80%)	284 (54.70%)
I would be WORRIED because my family and/or friends could not reach me	66 (12.70%)	59 (11.40%)	394 (75.90%)
I would feel NERVOUS because I would not be able to receive text messages and calls	105 (20.20%)	160 (30.80%)	254 (48.90%)
Section D: Giving up convenience			
If I did not have my smartphone with me-			
I would feel WEIRD because I would not know what to do	245 (47.20%)	149 (28.70%)	128 (24.70%)
I would feel the DESIRE to check it	100 (19.30%)	166 (32%)	253 (48.70%)
Section E: Daily habits			
I prefer to			
Use my smartphone until I fall asleep at night	117 (22.50%)	83 (16%)	319 (61.50%)
Wake up in the middle of the night to check notifications on my smartphone	402 (77.50%)	56 (10.80%)	61 (11.80%)
Check my smartphone immediately after I wake up in the morning	82 (15.8%)	127 (24.5%)	310 (59.7%)
Use my smartphone while I eat my meals	312 (60.10%)	82 (15.80%)	125 (24.10%)
Spend time on my smartphone during my break, as compared to doing any physical activity	95 (18.30%)	128 (24.70%)	296 (57%)
Watch movies/series on my smartphone for relaxation, as compared to exploring my hobbies	125 (24.10%)	138 (26.60%)	256 (49.30%)
Use my smartphone in the restroom/washroom/bathroom	126 (28.50%)	83 (16%)	310 (59.70%)
I am easily distracted by notifications on my smartphone and have difficulty resisting answering calls, texts, and emails	148 (28.50%)	186 (35.80%)	185 (35.60%)

Based on the modified NMP-Q, 387 (74.56%) of the respondents were reported to have moderate nomophobia, and 84 (16.18%) experienced severe nomophobia. Gender was associated with nomophobia level I and was statistically significant, with 63 (72.40%) females exhibiting severe levels as compared to 24 (27.60%) males, whereas 196 (54.70%) females had moderate levels as compared to only 162 (45.30%) males (p = 0.002). A similar pattern emerged for nomophobia level II, with 60 (71.40%) females having severe levels as compared to 24 (28.60%) males (p = 0.009). Age was associated with nomophobia level I, where 48 (55.20%) of the severe levels were aged 18-25 years, and 33 (37.90%) were aged 26-35 years (p = 0.008). A similar pattern was observed with the younger ones having severe nomophobia level II, where 40 (47.60%) were aged 18-25 years and 38 (45.20%) were aged 26-35 years (p = 0.022). Table 3 summarizes the associations between nomophobia and gender and age.

**Table 3:** Associations between nomophobia with age and gender.

Gender vs. nomophobia	Nomophobia Level I			P-value
	Absent/mild	Moderate	Severe	
Males	38 (53.40%)	162 (45.30%)	24 (27.60%)	0.002
Females	34 (46.60%)	196 (54.70%)	63 (72.40%)	
Gender vs. nomophobia	Nomophobia Level II			P-value
	Absent/mild	Moderate	Severe	
Males	23 (48.90%)	178 (46.0%)	24 (28.60%)	0.009
Females	24 (51.10%)	209 (54.0%)	60 (71.40%)	
Age vs. nomophobia	Nomophobia Level I			P-value
	Absent/mild	Moderate	Severe	
18-25	21 (28.80%)	169 (47.10%)	48 (55.20%)	0.008
26-35	34 (46.60%)	142 (39.60%)	33 (37.90%)	
36-45	14 (19.20%)	37 (10.30%)	4 (4.60%)	
≥46	4 (5.50%)	11 (3.10%)	2 (2.30%)	
Age vs. nomophobia	Nomophobia Level II			P-value
	Absent/mild	Moderate	Severe	
18-25	14 (29.80%)	184 (47.40%)	40 (47.60%)	0.022
26-35	20 (42.60%)	151 (38.90%)	38 (45.20%)	
36-45	9 (19.10%)	40 (10.30%)	6 (7.10%)	
≥46	4 (8.50%)	13 (3.40%)	0 (0.0%)	

## Discussion

In our study, most of the respondents (n = 238, 45.90%) were aged 18-25 years. Female participants were more than males (293 (56.50%) vs. 225 (43.40%)), and Android users were more than Apple iOS users (300 (57.80%) vs. 210 (40.50%)). In both personal and work-related activities, the maximum number of respondents spent less than three hours daily (336 (64.70%) and 426 (82.10%)). The prevalence of both moderate and severe nomophobia level II was found to be higher in females than in males. Among females, 24 (51.1%) had mild nomophobia (compared to 23 (48.9%) in males), 209 (54.0%) had moderate nomophobia (vs. 178 (46.0%) in males), and 60 (71.4%) had severe nomophobia (vs. 24 (28.6%) in males). A descriptive cross-sectional study conducted among dental students in India also found that the prevalence of nomophobia was higher among females (28.66%) than males (20.68%) [6]. Daily dependence on their smartphones was higher in female students (15.40%) than in male students (5.20%) in a cross-sectional study conducted among university students during the COVID-19 outbreak [1]. A systematic review of 108 studies by Leon-Mejia et al. reported a higher prevalence of nomophobia among females and young adults, consistent with the findings of our study. The majority of these studies reporting greater nomophobia among women were conducted across Asia, Europe, and the Americas. The diversity of cultures present in these regions indicates that cultural or social factors alone may not sufficiently account for the observed gender disparities [7].

Additionally, level II moderate and severe nomophobia were higher in the age group of 18-25 years. This finding is consistent with previous research indicating that individuals aged 18-25 years may be more susceptible to nomophobia [8,9]. There are several probable reasons for this finding. Firstly, young adults rely heavily on mobile phones to study and access medical information quickly. They use mobile apps and online resources for continuous learning and staying updated on the latest medical advancements. Secondly, smartphones play a vital role in social networking within the professional community, facilitating collaboration, knowledge-sharing, and discussions [10]. Thirdly, smartphones provide a convenient means to connect with friends and family for young adults staying away from home to study [11].

In our study, the percentage of respondents who strongly agreed or agreed that running out of battery in their smartphones would scare them, getting panicked if they ran out of credit or monthly data, and constantly checking for signals/Wi-Fi networks when unable to connect were 33.50% (n = 174), 20.80% (n = 108), and 48.70% (n = 254), respectively. These findings underscore the emotional responses associated with potential disruptions to smartphone functionality and connectivity, shedding light on the perceived importance of these devices in maintaining a sense of connection and identity. Additionally, the percentage of respondents who strongly agreed or agreed that they would feel anxious at the thought of not being able to communicate with family and friends instantly, and feeling nervous when unable to receive text messages and calls was 54.70% (n = 284) and 48.90% (n = 254), respectively. Similar to the findings of our study, another cross-sectional study among medical students found that 43.6% reported feeling impatient and fretful when they did not have their smartphone with them [9]. These findings highlight the emotional significance of smartphones for real-time communication, emphasizing the perceived impact on personal connections when access is hindered.

In 2010, Dixit et al. studied mobile phone dependence among medical graduates. The study found that 73% of the students kept their phones with them 24/7. Of the students, 20% reported feeling stressed when they did not have their phone or its battery was drained. Additionally, 38.50% of the students checked their phones repeatedly for messages and calls [12]. Our study also showed similar results, with 185 (35.60%) students strongly agreeing or agreeing that they get easily distracted by smartphone notifications and find difficulty resisting answering calls, texts, and emails. These findings align with those of a cross-sectional study conducted among nursing students, which reported an average smartphone-checking frequency of 8.0 ± 13.2 times per hour [13].

A cross-sectional study on nomophobia among anesthesiologists and intensivist professionals from 19 countries used a modified NMP-Q to determine nomophobia, as did our study. According to the survey, 33% had mild, 51% had moderate, and 15% had severe nomophobia [14]. According to the results of another cross-sectional study, mild, moderate, and severe nomophobia prevalence was found to be 13%, 52%, and 34%, respectively, among kidney specialists from 10 different countries. These studies suggest the magnitude of smartphone addiction among healthcare workers [15]. A recently published systematic review and meta-analysis based on 43 studies (n = 36,656 participants) about the global prevalence of nomophobia found that mild, moderate, and severe symptoms were reported by 26%, 51%, and 21% of participants [16]. Studies have shown that nomophobia is associated with stress disorder [2], poor academic performance [17], depression, anxiety, and poor quality of life [18]. Therefore, an improved understanding of its prevalence and determinants is needed to tackle this globally emerging issue.

Limitations

This cross-sectional study provided valuable information about the prevalence of nomophobia among the medical fraternity in Nepal. We acknowledge that there are a few limitations to our research. The sample size of 519 may not represent the diverse range of healthcare workers in Nepal, and the results may not be generalizable to the entire population or globally. Relying on self-reported data to assess nomophobia can introduce recall and social desirability biases. Participants may not accurately represent their smartphone dependency due to societal pressure. Moreover, voluntary random participation may create a selection and individual bias. This cross-sectional study may not account for changes over time or the dynamic nature of nomophobia prevalence among healthcare professionals. Conducting longitudinal studies with a more extensive and diverse group of participants can provide deeper insights into the patterns of nomophobia.

## Conclusions

This nomophobia study conducted in Nepal indicates a significant prevalence of nomophobia among specific demographic groups, particularly females and individuals aged 18-25 years, suggesting a higher tendency to become dependent on smartphones within these cohorts. It is not possible to establish causal relationships or track changes over time in this cross-sectional study. Future research involving a more significant number of medical professionals with diverse backgrounds should explore the underlying factors contributing to it.
